# Prevalence and Risk Factors of Vitamin D Deficiency in Patients Scheduled to Undergo Revision Arthroplasty of the Hip, Knee and Shoulder—Data from a Single-Centre Analysis

**DOI:** 10.3390/nu16183060

**Published:** 2024-09-11

**Authors:** Konstantin Horas, Miledi Hoxha, Tizian Heinz, Axel Jakuscheit, Kilian List, Gerrit S. Maier, Manuel Weißenberger, Maximilian Rudert

**Affiliations:** 1 Orthopaedic Center for Musculoskeletal Research, University of Wuerzburg, 97074 Wuerzburg, Germany; 2 Frankfurt Centre for Bone Health and Endocrinology, 60313 Frankfurt, Germany; 3 Department of Orthopaedic Surgery, Koenig-Ludwig-Haus, University of Wuerzburg, 97074 Wuerzburg, Germany; 4 Department of Orthopaedic Surgery, Pius-Hospital, Carl-von-Ossietzky-University, 26121 Oldenburg, Germany; 5 Orthopaedic Surgery Center, 97070 Wuerzburg, Germany

**Keywords:** vitamin D, hypovitaminosis D, revision arthroplasty, hip, knee, shoulder, periprosthetic joint infection, aseptic loosening

## Abstract

Vitamin D is crucial for ideal bone health and good muscle function, both essential requirements for successful joint arthroplasty. Hence, vitamin D deficiency has recently been identified as a predictor of poorer outcomes in patients scheduled to undergo total joint arthroplasty (TJA). Moreover, there is ample evidence today that vitamin D deficiency is associated with periprosthetic joint infection. Yet, vitamin D deficiency seems to be frequent in patients who are scheduled to undergo TJA. However, the prevalence of hypovitaminosis D in patients who require revision arthroplasty (rTJA) is largely unknown. Further, risk factors of vitamin D deficiency in these patients remain to be elucidated. For this reason, the primary objective of this study was to assess the vitamin D status of patients scheduled to undergo rTJA of the hip, knee and shoulder. The secondary objective was to identify potential risk factors for hypovitaminosis D in these patients. Serum vitamin D [25(OH)D] levels of 249 patients who were scheduled for rTJA were assessed over a period of twelve months at a high-volume TJA centre. Collectively, 23% of patients reported a routine intake of vitamin D supplements (58/249). Notably, 81% of patients (155/191) who did not report a routine vitamin D intake presented with insufficient vitamin D levels (below 30 ng/mL), while only 19% of patients (36/191) had sufficient vitamin D levels. Of those who reported a routine vitamin D intake, 75% (43/58) had sufficient vitamin D levels, while 25% (15/58) showed insufficient vitamin D status. Patients who did not routinely take any vitamin D supplements had significantly lower vitamin D levels compared to patients who reported regular vitamin D intake (19.91 ng/mL vs. 40.66 ng/mL). Further, BMI and nicotine abuse were identified as potential risk factors for hypovitaminosis D in patients without vitamin D supplementation. Moreover, the season of spring seems to be a risk factor in patients with vitamin D supplementation, while age itself did not appear to be a significant risk factor for low vitamin D levels. In conclusion, we found an alarmingly high rate of vitamin D deficiency in patients scheduled to undergo rTJA. Notably, reported routine vitamin D supplementation showed significantly increased serum vitamin D levels compared to patients with no reported supplementation. Due to the high prevalence of vitamin D deficiency, we believe that vitamin D status should routinely be assessed in patients who are scheduled to undergo rTJA.

## 1. Introduction

Modern hip and knee arthroplasty are surgical procedures with a high success rate of improving function and quality of life. Over recent decades, the number of procedures has increased steadily, and total joint arthroplasty (TJA) is now among the leading surgical procedures in Western countries [1,2]. Due to an ageing population, numbers are expected to rise even further. Forecasts predict an increase in total hip arthroplasty (THA) procedures of 176% by 2040 and 659% by 2060 in the United States (US) [2]. Due to the constant improvement of implants and surgical procedures, excellent implant survivorship and patient-reported outcomes are reported nowadays [3]. Nonetheless, as with any other surgical procedure, TJA is not without any risk. Hence, some patients might require a second operation on a previously replaced joint due to various indications. In this so-called revision arthroplasty, generally, at least one of the implanted components is exchanged, removed, added or modified.

Given the increasing number of primary TJA, revision rates have also risen continuously. Likewise, the projections show an increase in revision TJA (rTJA) procedures of 42% by 2040 and 101% by 2060 in the US [3]. The need for rTJA places a great burden not only on patients and surgeons but also on our already strained healthcare systems [4]. For this reason, it is of utmost importance to identify risk factors for the failure of primary TJA. Currently, common reasons for rTJA are mechanical failure with or without dislocation, infection, periprosthetic fracture or metallosis. Data from several joint registries show that aseptic loosening is a major reason for revision surgery. The process of aseptic loosening is oftentimes multifaceted and may occur because of inadequate fixation at the initial surgery or mechanical loss of fixation over time [5]. Further, it is believed that impaired local bone biology may contribute to aseptic loosening of the prosthesis. Hence, healthy bone is essential for successful TJA.

Vitamin D plays a key role in the maintenance of a healthy bone metabolism [6]. In particular, it is crucial in the regulation of serum calcium and phosphate, which are, in turn, needed for the normal mineralisation of bone. Homeostasis is achieved by the regulation of calcium and phosphate absorption and excretion and via the mobilisation or deposition of calcium or phosphate into bone. Resident osteoclasts continuously remove old or mechanically unnecessary bone, while osteoblasts replace this with newly formed bone. Under physiological conditions, removed bone is replaced by an equal amount of newly formed bone to protect the structural integrity of the skeletal system. This life-long continuum is susceptible, as bone remodelling not only comprises the complex interplay between osteocytes, osteoblasts and osteoclasts but is also affected by hormones and local growth factors [7]. In the case of persistently low vitamin D levels, this fragile balance is disrupted, resulting in enhanced bone turnover via the upregulation of the parathyroid hormone (PTH) and osteoclast activation [8]. 

Vitamin D deficiency is common and estimated to afflict more than one billion people globally [7]. Vitamin D is known to impact a variety of systems within the human body [9]. Moreover, several studies have linked vitamin D deficiency to poorer health outcomes in orthopaedic patients scheduled to undergo surgery, including TJA [10,11]. Vitamin D deficiency is also frequently seen in orthopaedic patients [12,13]. Further, its prevalence increases with age, thus coinciding with the rise in the incidence of TJA [12]. It is, therefore, not surprising that low vitamin D levels have also been reported in patients scheduled to undergo TJA [13,14,15].

At present, there is increasing evidence that impaired bone metabolism, as seen in vitamin D deficiency, is of relevance for patients undergoing TJA. However, there is currently hardly any data on the prevalence and role of vitamin D deficiency in TJA, especially in rTJA. Thus, the primary objective of this study was to analyse the vitamin D status of patients scheduled to undergo rTJA. Concomitantly, our secondary objective was to identify potential risk factors for hypovitaminosis D in this patient cohort.

## 2. Patients and Methods

Over a period of 12 months (May 2023–May 2024), serum 25(OH)D, which is a reliable marker of vitamin D status, of 249 patients who were scheduled for rTJA of either the hip, knee or shoulder were measured. Moreover, serum PTH and calcium levels were assessed. All measurements were conducted at the Department of Orthopaedics, Koenig-Ludwig-Haus, University of Wuerzburg, Germany (49.76° N latitude). Inclusion criteria were the need for revision arthroplasty of either the hip, knee or shoulder and being 18 years of age or older. There were no restrictions with regard to gender, weight or the number of previous surgeries on the affected joint. All patients had to complete a detailed questionnaire on their medical history and provide a medication chart. Further, all patients were interviewed to discuss their medical history and routine medication, including vitamin D supplementation. Patients with known diseases that could potentially impact on their vitamin D, PTH or calcium status, such as general bone metabolism disorders, were excluded. Moreover, we excluded all patients who reported a routine intake of osteoporosis medications. Patients with any other or no routine medications or comorbidities were eligible for inclusion. 

All experiments were conducted in accordance with the guidelines of the Committee of Medical Ethics and the World Medical Association Declaration of Helsinki (Ethics number 1/23-me). Prior to the collection of venous blood samples, approval was obtained from every patient. Blood samples were generally collected the day before surgery. A standardised method for serum measurements using the cobas^®^ 25-Hydroxyvitamin D Assay (Vitamin D Total) and the Elecsys PTH (1–84) assay for cobas^®^ e411 Analyzer (Roche Diagnostics, Mannheim, Germany) was applied. All laboratory results and patient data were collected using a retrospective chart review.

Additionally, patients were screened for risk factors for osteoporosis according to the guidelines of the German Osteology Society (DVO). Screening included body mass index (BMI), comorbidities (e.g., smoking and neurological disorders) and routine medication intake (e.g., glucocorticoids or PPI). As vitamin D is mostly formed in the skin under the influence of sunlight, we also assessed the date of blood collection and grouped patients according to daily sun exposure in Germany into 4 cohorts (spring = March–May; summer = June–August; autumn = September–November; winter = December–February).

At present, there is still no international consensus on the levels of serum 25(OH)D regarded as sufficient, deficient or insufficient [7]. For data analysis and interpretation, we obeyed the guidelines of the US Endocrine Society (25(OH)D levels < 20 ng/mL (50 nmol/L) = vitamin D deficiency; 25(OH) D levels between 20 and 29 (50–72.5 nmol/L) = vitamin D insufficiency; and 25(OH)D levels ≥ 30 ng/mL (75 nmol/L) = vitamin D sufficient) [8]. All patients with valid 25(OH)D levels were included in the statistical analysis and grouped according to gender, age, season, BMI and smokers or non-smokers. Moreover, the reason for revision surgery (aseptic loosening, periprosthetic or deep wound infection, periprosthetic fracture, or wear out) was assessed. 

Statistic calculations were performed using GraphPad Prism statistical software (GraphPad Prism version 10.0.0, GraphPad Software, Boston, MA, USA). Categorical data were analysed using absolute and relative frequencies. The normal distribution of the data was evaluated using the Kolmogorov–Smirnov and Shapiro–Wilk tests. Based on these evaluations, either parametric or non-parametric tests were employed as appropriate. The mean serum levels of vitamin D, PTH and calcium were compared between groups using ANOVA and independent *t*-tests or their non-parametric counterparts when indicated. The correlative associations were assessed through linear and logistic regression analyses, including bivariate correlation testing. A *p*-value of less than 0.05 was considered statistically significant.

## 3. Results

In total, 249 patients who were scheduled to undergo revision surgery were enrolled in this study (*n* = 118 hip, *n* = 116 knee and *n* = 15 shoulder revision). A total of 142 patients were female (57%), and 107 patients were male (43%), with a cumulative mean age of 68.29 years (range of 26–96 years). Of these, 58 patients (23%) reported a routine intake of vitamin D supplements, while 191 patients (77%) did not take any vitamin D supplements. Of those who reported a routine intake of vitamin D supplements, 85% (*n* = 49) were female and 15% (*n* = 9) were male. It was found that 26% of patients (31/118) who were scheduled for revision surgery of the hip, 21% of patients (24/116) who were scheduled for revision surgery of the knee and 20% of patients (3/15) who were registered for revision surgery of shoulder arthroplasty reported a routine intake of vitamin D supplements. Collectively, 84 patients presented with a periprosthetic infection (*n*= 40 for hip, *n* = 34 for knee and *n* = 10 for shoulder; 34% of all patients) and 5 patients with periprosthetic fractures (all hip, 2% of all patients), while 160 patients (*n* = 50 for hip, *n* = 82 for knee and *n* = 5 for shoulder; 64% of all patients) were scheduled to undergo revision surgery due to aseptic loosening or another complication associated with the implant (Table 1).

**Figure 1:**
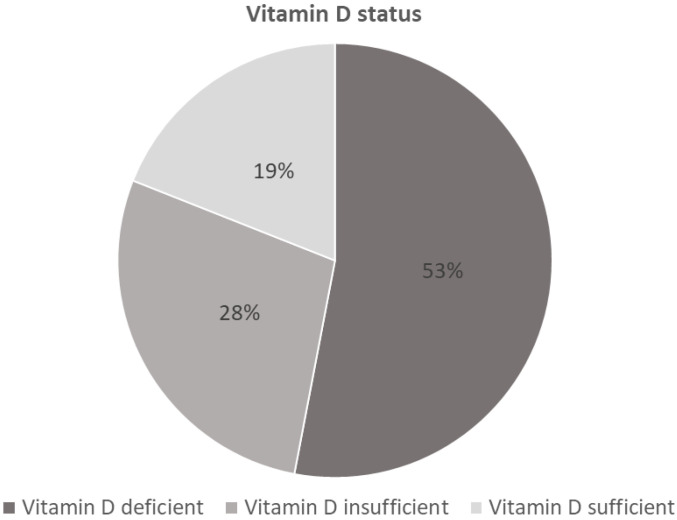
Vitamin D status of patients who did not report a routine vitamin D intake (*n* = 191).

Altogether, 81% of patients (155/191) who did not report a routine vitamin D intake, presented with low vitamin D levels (*n* = 102 [53%] vitamin D deficient; *n* = 53 [28%] vitamin D insufficient), while 19% of patients (36/191) had sufficient vitamin D levels (Figure 1). 

**Figure 2:**
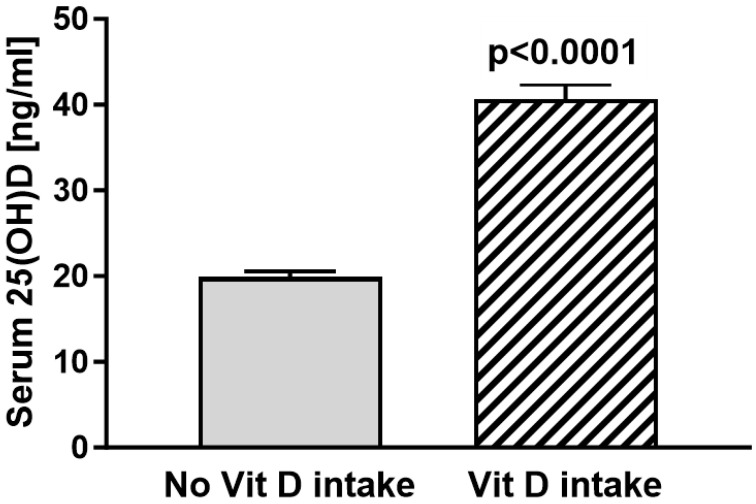
Serum 25(OH)D levels of patients without routine vitamin D intake [mean 19.91 ng/mL; *n* = 191] were significantly lower compared to patients with routine vitamin D intake [mean 40.66 ng/mL (55.4; *n* = 58)] (*p* < 0.0001). Results are shown as mean ± SEM.

Of those that reported a routine vitamin D intake, 74% (43/58) had sufficient vitamin D levels, while 25% (15/58) showed insufficient vitamin D levels (*n* = 1 vitamin D deficient; *n* = 14 vitamin D insufficient). The total mean vitamin D levels were 19.91 ng/mL (29.9 nmol/L) in the cohort without routine vitamin D intake and 40.66 ng/mL (61.0 nmol/L) in the routine vitamin D intake group (Figure 2). The statistical analyses of vitamin D levels in patients without routine vitamin D intake compared to the vitamin D levels of patients with routine vitamin D intake show significantly lower vitamin D levels in patients without routine vitamin D intake (19.91 vs. 40.66 ng/mL *p* < 0.0001) (Figure 2).

**Figure 3:**
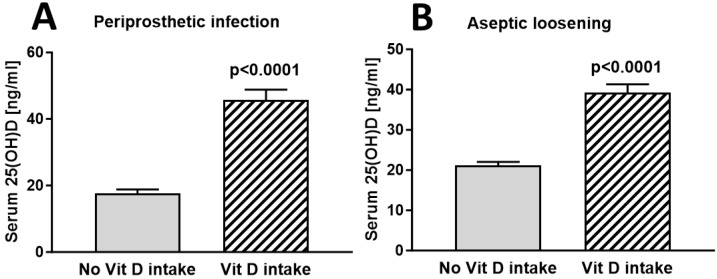
Serum 25(OH)D levels of patients with periprosthetic infection grouped according to routine vitamin D intake (**A**) (*n* = 69 for no routine vitamin D intake and *n* = 15 for routine vitamin D intake, 17.67 ng/mL vs. 44.80 ng/mL) and aseptic loosening (**B**) (*n* = 117 for no intake and *n* = 43 for routine intake, 21.20 ng/mL vs. 39.21 ng/mL). Results are shown as mean ± SEM. Mean vitamin D level of patients who presented with periprosthetic fractures (*n* = 5) was 20.50 ng/mL.

For an additional comparison, we further subdivided cohorts according to diagnosis and vitamin D intake. Patients who were diagnosed with periprosthetic infection and no routine vitamin D intake (*n* = 69) showed a mean vitamin D level of 17.67 ng/mL compared to 45.71 ng/mL (*n* = 14) (*p* < 0.001) with reported routine vitamin D intake (Figure 3A). A total of 62% of patients with no routine vitamin D intake were vitamin D deficient (42/69), 23% were vitamin D insufficient (16/69), and merely 15% had sufficient vitamin D levels (10/69). Patients who presented with aseptic loosening and no routine vitamin D intake (*n* = 117) showed a mean vitamin D level of 21.20 ng/mL compared to 39.21 ng/mL (*n* = 43) (*p* < 0.001) with reported routine vitamin D intake (Figure 3B). In particular, 49% were vitamin D deficient (56/117), 30% were vitamin D insufficient (36/117), and 21% had sufficient vitamin D levels (25/117). Comparing the mean vitamin D levels of patients with periprosthetic infections to patients with aseptic loosening, no significant difference was found (*p* = 0.682). We further analysed the data to determine whether vitamin D supplementation influences the incidence rates of periprosthetic infection and aseptic loosening. Chi-square testing found no statistically significant association between vitamin D supplementation and the incidence of aseptic loosening or periprosthetic infection (X2 = 2.04; *df* = 2; *p* = 0.36). To further investigate a potential association between vitamin D status and the usage of either cemented or non-cemented implants, we further subdivided the cohort of aseptic loosening. There were no apparent differences in the vitamin D status of patients who were scheduled for revision surgery of the hip or shoulder and received cemented arthroplasty without vitamin D supplementation (*n* = 13, mean vitamin D = 23 and 53 ng/mL) compared to those who received non-cemented revision arthroplasty (*n* = 31, mean vitamin D = 21 and 20 ng/mL). The vitamin D status of patients who reported routine vitamin D supplementation generally had increased vitamin D levels (*n* = 8, mean vitamin D = 33 and 25 ng/mL for cemented revision arthroplasty; *n* = 11, mean vitamin D 43 and 10 ng/mL for non-cemented revision arthroplasty). Due to the small patient numbers and high heterogeneity within the cohort, we omitted statistical tests of significance.

**Figure 4:**
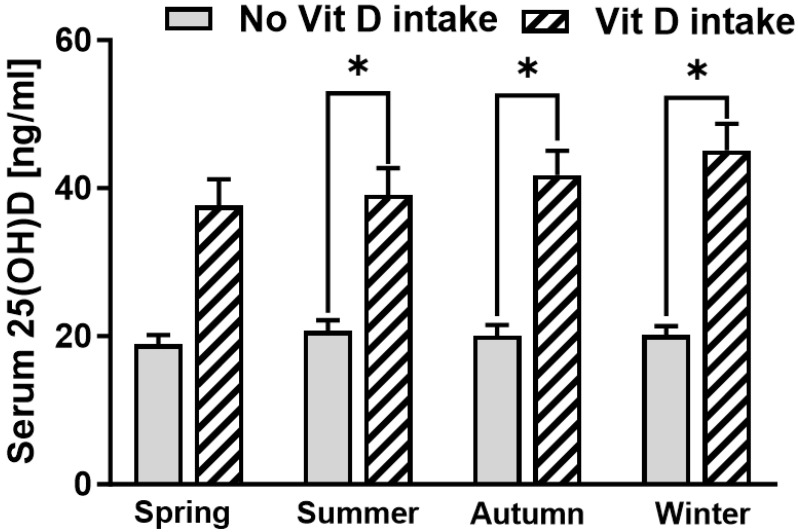
Serum 25(OH)D levels of patients with and without routine vitamin D intake in correlation to the season [spring (March–May): *n* = 54 no intake (19.08 ng/mL) and *n* = 14 (39.07 ng/mL) for intake; summer (June–August): *n* = 44 no intake (20.70 ng/mL) and *n* = 12 (41.75 ng/mL) for intake; autumn (September–November): *n* = 40 no intake (20.05 ng/mL) and *n* = 15 (45.13 ng/mL) for intake; winter (December–February): *n* = 53 no intake (20.15 ng/mL) and *n* = 16 (37.69 ng/mL) for intake]. Asterisk marks significant differences in serum 25(OH)D (*p* < 0.05).

As a seasonal variation in vitamin D status has frequently been reported, we analysed for differences in vitamin D levels depending on the season. The serum vitamin D levels in patients without routine vitamin D intake were low in all seasons (Figure 4). 

Overall, 144 (58%) patients reported a regular intake of drugs that may induce osteoporosis (according to the DVO guidelines). The most frequent medication was the chronic intake of proton-pump inhibitors (PPIs) in 96 (39%) patients. Notably, 26 patients (10%) reported a chronic glucocorticoid intake. We also screened for comorbidities that are considered risk factors for osteoporosis, according to the DVO. Notably, 85 patients (34%) had such comorbidities; among the most frequent were diabetes (38 patients), nicotine dependence (27 patients) and chronic obstructive pulmonary disease (COPD, 13 patients). Ten patients without routine vitamin D intake had increased PTH levels. All of them demonstrated secondary hyperparathyroidism due to severe vitamin D deficiency. Serum calcium was low in four patients. All of them had low vitamin D and increased PTH levels. In the vitamin D supplementation cohort, only one patient had an increased PTH level. All serum calcium levels were within the normal range. 

Analysing risk factors for hypovitaminosis D, we found significantly lower vitamin D levels in smokers (*n* = 27, 15.81 ng/mL) compared to non-smokers (*n* = 198, 25.23 ng/mL) (*n* = 24, no data on smoking status). Further, obese patients (BMI > 30) with no reported vitamin D supplementation had particularly low vitamin D levels (mean 10 ng/mL, *n* = 6) compared to obese patients with routine vitamin D intake (39, 48 ng/mL, *n* = 25). Moreover, vitamin D levels were lower in males (mean 20.97 ng/mL, *n* = 107) compared to females (27.65 ng/mL, *n* = 142). Interestingly, age analysis reveals that patients above the age of 70 years without a routine intake of vitamin D (*n* = 88) had higher mean vitamin levels compared to patients below the age of 70 years without a routine vitamin D intake (*n* = 103, 20.47 vs. 19.42). Notably, the mean vitamin D levels of patients above the age of 70 who reported routine vitamin supplementation presented with a mean vitamin D level of 41.69 ng/mL (*n* = 26). The risk factors for hypovitaminosis D are further demonstrated in Table 2. 

## 4. Discussion

Findings from the current study show an alarmingly high rate of vitamin D deficiency in patients scheduled to undergo rTJA. It has become evident in recent years that vitamin D deficiency is common in orthopaedic patients [16]. Concomitantly, several studies have shown a high prevalence of vitamin D deficiency in patients scheduled to undergo hip, knee and shoulder arthroplasty [13,14,17,18]. In a recent meta-analysis on hypovitaminosis D in lower extremity joint arthroplasty by Emara et al. the authors reported a pooled prevalence of vitamin D insufficiency and deficiency of 53.4% and 39.4%, respectively [17]. Similar results have been reported for total shoulder arthroplasty. In a single-centre analysis by Inkrott et al. 43% of patients were vitamin D insufficient, and a further 11% were vitamin D deficient [19]. Another very recent examination by MacConnell et al. identified 29.79% of shoulder arthroplasty patients as vitamin D insufficient and another 35.11% as being vitamin D deficient [20]. Data on the prevalence of vitamin D deficiency in rTJA are very rare. In a retrospective review of 126 revision total joint arthroplasties by Traven et al., the reported prevalence of vitamin D deficiency among rTJA patients was 55% [21]. Another retrospective cohort analysis of 20 patients who underwent 23 revision total knee arthroplasty procedures identified 57% of patients as vitamin D deficient [22]. This is in line with our findings, with 53% of patients (without routine vitamin D intake) being vitamin D deficient and 28% being vitamin D insufficient. 

Today, it has become evident that low pre-operative vitamin D levels may increase the risk of postoperative complications, including periprosthetic joint infection, in patients undergoing primary TJA [10,17]. In a recent meta-analysis, Vivek et al. concluded that vitamin D deficiency leads to poorer health outcomes and greater length of stay after total knee arthroplasty [11]. Similarly, vitamin D deficiency is associated with a higher rate of all-cause revision total shoulder arthroplasty [18]. It is, therefore, not surprising that some authors suggest that the pre-operative optimisation of vitamin D levels may be beneficial in reducing postoperative complications [10,23]. It was demonstrated that administering an oral single dose of 300,000 IU vitamin D is able to correct vitamin D deficiency and has a positive impact on health outcomes following primary TJA [10]. Another treatment regimen suggests a loading dose of 50,000 IU weekly for 4 weeks, followed by a maintenance dose of 2000 IU/d among total knee arthroplasty patients [24]. Although some studies have demonstrated a positive effect of pre-operative vitamin D supplementation on outcomes in TJA patients, others have failed to support such an association [10,11,20,25]. Thus, further adequately powered randomised, controlled trials using vitamin D supplementation are required to assess if vitamin D deficiency is a modifiable risk factor to improve outcomes in TJA [26]. To the best of our knowledge, there is currently only one study reported in the literature investigating the outcome of patients following rTJA in association with vitamin D status. Traven et al. found fewer complications following revision hip and knee arthroplasty in patients with normal vitamin D levels [21]. If this also accounts for patients scheduled to undergo revision shoulder arthroplasty has yet to be elucidated. 

Collectively, recent studies suggest that vitamin D deficiency is frequent in patients scheduled to undergo TJA. Furthermore, low vitamin D levels might be associated with worse outcomes in TJA patients. In the current study, we found a widespread and alarming rate of vitamin D deficiency and insufficiency among patients scheduled for rTJA. To date, there is a plethora of studies, some of which date back twenty years or longer, that demonstrate the potential health-beneficial effects of vitamin D not only on bone but also on muscle function and the immune system [6,8,9,27]. In addition, several studies demonstrate a high prevalence of vitamin D deficiency in orthopaedic patients, including patients scheduled to undergo TJA [13,14,18]. It was, therefore, somewhat surprising to see that in the current study, only 23% of patients reported a routine intake of vitamin D supplements. Hence, only 20% of patients who did not report routine intake of vitamin D supplements presented with sufficient vitamin D levels. Further, most patients who reported a routine vitamin D intake had sufficient vitamin D levels. For this reason, vitamin D supplementation seems to be beneficial for patients scheduled for TJA and rTJA. Thus, we believe that vitamin D status should routinely be assessed in patients scheduled to undergo rTJA.

Healthy bone can be regarded as a conditio sine qua non for successful cementless THA [28]. In osteopenia or osteoporosis, patients not only have low bone mineral density (BMD) but also thin, weak and brittle bones. Therefore, many orthopaedic surgeons generally prefer hybrid or fully cemented hip arthroplasty in these cases [29]. However, there seems to be a substantial portion of patients with osteoarthritis of the hip that have occult osteoporosis and hypovitaminosis D [15,30,31,32]. Notably, Wang et al. recently demonstrated that among high-risk patients undergoing TJA, 90% did not receive any pharmacological osteoporosis treatment. In particular, 45% of patients did not routinely take any vitamin D or calcium supplements. Apart from these observations, 88% did not receive any BMD testing [33]. Interestingly, even among patients with pre-existing osteoporosis undergoing TJA, 80% were not treated with any osteoporosis medications, and 33% of these patients were not taking vitamin D or calcium supplements [33]. In the current study, 34% of patients had comorbidities, and a remarkable 58% of patients reported routine medication intake that is considered a risk factor for osteoporosis. Furthermore, we identified smoking as a potential risk factor for hypovitaminosis D, which is also reported as a risk factor for osteoporosis. The exact mechanism by which smoking impacts vitamin D status is mostly unknown and has recently been discussed in detail (see [34]). 

As vitamin D is tightly bound in fatty tissues, several studies have reported an association between vitamin D deficiency and obesity [35]. In the current study, we did not identify any significant correlation between vitamin D levels and BMI. Vitamin D status has also been reported to be influenced by seasonal variation [12]. As daily sunshine hours in Germany are lowest in winter and spring, serum vitamin D levels have previously been reported to be lowest during these seasons [12,28]. In line with these observations, the risk of vitamin D insufficiency or deficiency was 2.23 times increased during spring compared to summer in the current study. Together, we believe that there should be an increased awareness among orthopaedic surgeons that patients scheduled to undergo rTJA not only often present with low vitamin D levels but also commonly have risk factors for osteoporosis. This is of high clinical relevance as many orthopaedic surgeons are known to adapt their surgical plan if osteoporosis or low BMD is known prior to surgery [28,36]. Moreover, it is conceivable that vitamin D deficiency might also play a role in the outcome of TJA; thus, patients with low vitamin D levels are more likely to undergo rTJA. Nonetheless, the reported association does not prove any causal relationship, and further studies, especially robust data, are needed to confirm these results.

We are aware that this investigation has several limitations. For example, the data shown in this study represent single-centre patient data of a geographical localisation of Wuerzburg (Bavaria, Germany [49°47′ N latitude]). Hence, patient data can only be compared to the serum vitamin D levels of patients living in latitudes comparable to that of Wuerzburg (e.g., Paris [48°51′ N], Vancouver [49°15′ N] and Kiev [50°27′ N]). Moreover, we do not have any robust data on the exact vitamin D intake of every patient. Data analysis relies on patient reports and the medical history of patients. Furthermore, we do not have data on the vitamin D status of patients with previously implanted joint arthroplasty without any evidence of periprosthetic infection or aseptic loosening. As such, without a suitable control group, this investigation must be regarded as a purely observational study, and no causal relationship between vitamin D status and patient outcomes can be drawn. 

In summary, we found a high prevalence of vitamin D deficiency in patients scheduled to undergo rTJA of the knee, hip and shoulder. In addition, numerous patients had risk factors for osteoporosis. Thus, we recommend the pre-operative assessment of the vitamin D status of these patients. Further, it should be considered that vitamin D supplementation is widely available, convenient, safe and effective in raising vitamin D levels. However, whether TJA patients with low vitamin D levels have a higher risk of revision surgery, in general, and, thus, would profit from vitamin D supplementation will need to be addressed in future studies.

## 5. Conclusions

In this single-centre analysis, we identified an alarming rate of vitamin D deficiency in patients scheduled to undergo rTJA. Although numerous studies have demonstrated the potential health-beneficial effects of vitamin D in orthopaedic patients, only very few patients seem to routinely take any vitamin D supplements. For this reason, we recommend that vitamin D levels should generally be assessed in these oftentimes frail and vulnerable patients.

## Figures and Tables

**Table 1:** Patient characteristics.

		Number of Patients		
			Vit D Suppl. (Total Number; Percentage)	
		Total (249; 100%)	No. (191; 77%)	Yes (58; 23%)
Gender	Female	142 (57%)	93 (65%)	49 (35%)
	Male	107 (43%)	98 (92%)	9 (8%)
Revision	Hip	118	87 (74%)	31 (26%)
	Knee	116	92 (79%)	24 (21%)
	Shoulder	15	12 (80%)	3 (20%)
Reason	Infection	84 (34%)	69 (82%)	15 (18%)
	As. Loos.	160 (64%)	117 (73%)	43 (27%)
	Fracture	5 (2%)	5 (100%)	0
Age	Range: 26–96 years		Mean: 68.29 years	

**Table 2:** Analysis of risk factors for hypovitaminosis D in patients without vitamin D supplementation (A) and with vitamin D supplementation (B). Age (range 26–96 years); BMI (not categorised); nicotine abuse (yes or no); season (spring, summer, autumn, winter).

**(A)** Patients without oral supplementation						
**Predictor**	**Coefficient**	***n***	**Std. Error**	**T-Statistic**	***p*-Value**	**95% CI**
Sex Male vs. Female	1.66	98/98	1.47	1.12	0.26	−1.26; 0.47
Age	−0.02	196	0.08	−0.23	0.82	−0.17; 0.14
BMI	−0.36	196	0.12	−2.98	<0.01	−0.12; −2.98
Nicotine abuse	−7.51	21	2.47	−3.04	<0.01	−12.38; −2.64
Season	0.04	196	0.61	0.07	0.95	−1.17; 1.25
**(B)** Patients with oral supplementation						
**Predictor**	**Coefficient**	***n***	**Std. Error**	**T-Statistic**	***p*-Value**	**95% CI**
Sex Male vs. Female	2.65	9/48	4.98	0.53	0.59	−13.27; 10.62
Age	0.28	57	0.26	1.09	0.28	−0.24; 0.81
BMI	−0.01	57	0.03	−0.01	0.99	−0.62; 0.62
Nicotine abuse	−1.33	6	5.91	−0.22	0.82	−13.27; 10.62
Season	3.97	57	1.74	2.28	0.03	0.45; 7.49

## Data Availability

The original contributions presented in the study are included in the article, further inquiries can be directed to the corresponding author. The data are not publicly available due to privacy.
